# Multi‐omic analysis of biological aging biomarkers in long‐term calorie restriction and endurance exercise practitioners: A cross‐sectional study

**DOI:** 10.1111/acel.14442

**Published:** 2024-12-18

**Authors:** Giovanni Fiorito, Valeria Tosti, Silvia Polidoro, Beatrice Bertozzi, Nicola Veronese, Edda Cava, Francesco Spelta, Laura Piccio, Dayna S. Early, Daniel Raftery, Paolo Vineis, Luigi Fontana

**Affiliations:** ^1^ Clinical Bioinformatics Unit IRCCS Istituto Giannina Gaslini Genoa Italy; ^2^ Department of Medicine Washington University School of Medicine St. Louis Missouri USA; ^3^ Department of Translational Medicine (DiMET) CAAD Center for Translational Research on Autoimmune and Allergic Disease University of Eastern Piedmont "Amedeo Avogadro" Novara Italy; ^4^ Geriatric Unit, Department of Internal Medicine and Geriatrics University of Palermo Palermo Italy; ^5^ Unit of Dietetic and Clinical Nutrition, San Camillo Forlanini Hospital Rome Italy; ^6^ Geriatric Unit, AULSS 9 Scaligera “Mater Salutis” Hospital, Legnago Verona Italy; ^7^ Brain and Mind Centre, Faculty of Medicine and Health University of Sydney Sydney New South Wales Australia; ^8^ Northwest Metabolomics Research Center, Department of Anesthesiology and Pain Medicine University of Washington Washington USA; ^9^ MRC‐PHE Centre for Environment and Health Imperial College London London UK; ^10^ Charles Perkins Center, Faculty of Medicine and Health University of Sydney Sydney New South Wales Australia; ^11^ Department of Endocrinology Royal Prince Alfred Hospital Sydney Australia

**Keywords:** biological aging, calorie restriction, multi‐omic, oxidative stress, physical exercise

## Abstract

Calorie restriction (CR) and physical exercise (EX) are well‐established interventions known to extend health span and lifespan in animal models. However, their impact on human biological aging remains unclear. With recent advances in omics technologies and biological age (BioAge) metrics, it is now possible to assess the impact of these lifestyle interventions without the need for long‐term follow‐up. This study compared BioAge biomarkers in 41 middle‐aged and older adult long‐term CR practitioners, 41 age‐ and sex‐matched endurance athletes (EX), and 35 sedentary controls consuming Western diets (WD), through PhenoAge: a composite score derived from nine blood‐biomarkers. Additionally, a subset of participants (12 CR, 11 EX, and 12 WD) underwent multi‐omic profiling, including DNA methylation and RNAseq of colon mucosa, blood metabolomics, and stool metagenomics. A group of six young WD subjects (yWD) served as a reference for BioAge calculation using Mahalanobis distance across six omic layers. The results demonstrated consistently lower BioAge biomarkers in both CR and EX groups compared to WD controls across all layers. CR participants exhibited lower BioAge in gut microbiome and blood‐derived omics, while EX participants had lower BioAge in colon mucosa‐derived epigenetic and transcriptomic markers, suggesting potential tissue‐specific effects. Multi‐omic pathway enrichment analyses suggested both shared and intervention‐specific mechanisms, including oxidative stress and basal transcription as common pathways, with ether lipid metabolism uniquely enriched in CR. Despite limitations due to sample size, these findings contribute to the broader understanding of the potential anti‐aging effects of CR and EX, offering promising directions for further research.

## Graphical abstract

We observed lower biomarkers of biological aging in individuals practicing calorie restriction (CR) and endurance exercise runners (EX) compared to sedentary controls following a Western diet (WD), based on multiple omic analyses. These findings support recent epidemiological findings about the beneficial effect of CR and EX, suggest potential tissue‐specific effects, and highlight the reduction of oxidative stress damage as a key mechanism underlying the anti‐aging benefits of both CR and EX.

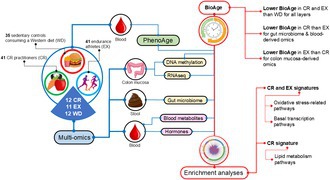

## 1 INTRODUCTION

Calorie restriction (CR) and endurance exercise (EX) have emerged as effective strategies for preventing chronic diseases across a range of organisms, including humans. In animal models, these interventions prolong maximal lifespan, prevent or delay age‐related diseases onset, and enhance cognitive and functional performance (Green et al., 2022; Levine et al., 2020; Maegawa et al., 2017). However, the long‐term effects of CR and EX on slowing human biological aging are still not fully understood.

Recent advancements in omics technologies, coupled with the development of biological age (BioAge) algorithms based on molecular biomarkers, provide a unique opportunity to assess the potential benefits of different lifestyle or pharmacological interventions without the need for prolonged follow‐up periods typically required in epidemiological studies (Rutledge et al., 2022). Accumulating evidence from prospective studies suggest that these biomarkers can effectively gauge biological aging and predict longevity and the risk of age‐related diseases (Erema et al., 2022; Kuiper et al., 2023). Cross‐sectional observational studies examining molecular biomarkers of biological aging offer valuable insights into the potential benefits of long‐term (>3 years) CR and endurance EX, overcoming the nearly insurmountable challenges of multiyear clinical trials, such as adherence and cost.

Various BioAge estimation methods have emerged, including DNA methylation (DNAm) epigenetic clocks, metabolomics‐based aging clocks, telomere length analysis, and phenotypic age (PhenoAge) assessments using blood biomarkers (Horvath, 2013; Levine et al., 2018; Robinson et al., 2020). New statistical approaches, such as Cohen's method, allow BioAge to be computed as a multivariate distance from a reference young population, addressing batch effects and capturing nonlinear biomarker relationships (Cohen et al., 2013; Kwon & Belsky, 2021).

This pilot study explores BioAge differences among middle‐aged and older adults (ages 34–82, average 55) who practice long‐term CR without malnutrition or endurance EX, compared to age‐ and sex‐matched healthy individuals consuming a Western Diet (WD). BioAge was calculated using multiple omic biomarkers, including proteins and immune biomarkers from Levine's PhenoAge, gut microbiome data, blood metabolomics and hormone profiles, and DNAm and transcriptomics from colon mucosa biopsies. A group of six young (ages 21–27, average age 24) men and women consuming Western diets (yWD) served as the reference population.

## 2 METHODS

### 2.1 Study sample description

The study comprises 41 lean men and women practicing CR for an average of ∼7 years (range, 3–15 years), along with 41 age‐ and sex‐matched endurance runners (EX) on a high‐calorie diet, and 35 age‐ and sex‐matched sedentary controls following a Western diet (WD). The energy intake was: 1779 ± 355 kcal/day for the CR group; 2433 ± 502 kcal/day for the WD group; and 2811 ± 711 kcal/day for the EX group. Additionally, six young nonobese individuals, aged 21–27 years, consuming WD (yWD), served as the reference group for computing biological age (BioAge) measures. Stringent selection criteria ensured fair and unbiased comparisons among groups. All participants reported weight stability (within 2 kg) over the preceding 6 months, were free of chronic disease, nonsmokers, and did not use medications affecting study outcomes. Educational levels were comparable across groups. Participant characteristics are summarized in Table 1, and additional information on the study sample characteristics are provided in the Data S1. This study was approved by the Human Studies Committee of Washington University School of Medicine, and all subjects gave informed consent before their participation.

**TABLE 1:** Description of the study sample. Continuous variables are described with their mean, standard deviation, and range; categorical variables are described as absolute numbers and percentages; *p‐values refer to the ANOVA test for continuous variables and the Chi‐squared test for categorical variables.

	Study sample					Subset for multi‐omic analyses				
	CR (*N* = 41)	EX (*N* = 41)	WD (*N* = 35)	yWD (*N* = 6)	p*	CR (*N* = 12)	EX (*N* = 11)	WD (N = 12)	yWD (N = 6)	p*
Age					0.212					0.83
Mean (SD)	54.2 (11.6)	56.0 (10.3)	58.5 (9.6)	24.3 (2.0)		61.7 (8.4)	58.6 (8.3)	62.4 (8.5)	24.3 (2.0)	
Range	34; 82	42; 78	37; 74	21; 27		48; 73	50; 73	46; 74	21; 27	
Sex					0.428					1
Males	36 (88%)	35 (85%)	27 (77%)	4 (67%)		10 (83%)	9 (82%)	10 (83%)	4 (67%)	
Females	5 (12%)	6 (15%)	8 (23%)	2 (33%)		2 (17%)	2 (18%)	2 (17%)	2 (33%)	
BMI kg/m^2^					<0.001					<0.001
Mean (SD)	19.2 (1.4)	22.7 (2.4)	25.5 (3.5)	25.7 (0.9)		19.1 (1.4)	24.5 (2.8)	27.4 (2.5)	25.7 (0.9)	
Range	16.5; 22.8	17.6; 28.4	18.0; 31.5	24.4; 27.2		17.3; 21.3	20.7; 28.4	24.2; 31.5	24.4; 27.2	
Body fat %					<0.001					<0.001
Mean (SD)	9.5 (6.1)	14.8 (6.6)	25.9 (6.8)	17.9 (8.2)		13.4 (5.5)	19.4 (5.3)	28.3 (8.6)	17.9 (8.2)	
Range	0.2; 26.2	1.5; 28.0	15.0; 48.4	7.3; 29.3		6.0; 25.4	10.7; 28.0	17.0; 48.4	7.3; 29.3	

Blood‐measured and immune system‐related biomarkers composing the PhenoAge algorithm, developed by Levine et al. (Levine et al., 2018), were measured across the entire sample. A subset of 12 CR, 11 EX, 12 WD, and 6 yWD participants underwent comprehensive multi‐omic profiling, including DNAm (Illumina 450 K BeadChip array) and transcriptomic (RNAseq) profiling from colon mucosa biopsies, stool metagenomics (gut microbiota 16S rRNA sequencing), plasma metabolomics (targeted LC–MS/MS), and hormonal assessment (combination of ELISA and various Luminescent Assays). Detailed methodologies can be found in the Data S1.

### 2.2 Computation of multi‐omic BioAge biomarkers and association analyses

For each omic layer, we calculated corresponding BioAge measures, resulting in six distinct biomarkers: phenoBioAge, microbiomeBioAge, metabolomeBioAge, hormoneBioAge, DNAmBioAge, and transcriptomeBioAge. These BioAge measures are defined using Mahalanobis distance relative to the average profile of the young reference group (yWD), as implemented in the *BioAge* R package (Kwon & Belsky, 2021). By utilizing a young reference population, we ensured precise comparisons across diverse omic profiles, addressed the lack of standardized definitions for some BioAge measures (e.g., gut microbiome), and mitigated bias induced by batch effects. The standardization of these six BioAge measures accounted for variations in the number of features per omic layer, allowing for meaningful comparisons. Values close to 0 indicate omic profiles similar to the average young reference individual (younger BioAge), while higher values indicate older BioAge. Comparisons of BioAge values among the three adult groups were performed using two‐tailed pairwise t‐tests, with Tukey HSD adjustments for multiple comparisons, setting *α* = 0.05 as the significance threshold.

### 2.3 Identification of multi‐omic CR and EX signatures

Following quality control and multi‐omics data preprocessing, our dataset included six omic layers: 16S rRNA gene gut microbiome data (1321 OTUs), DNAm (346,699 CpG sites) and mRNA (23,562 genes) data from colon mucosa, plasma metabolomics (108 metabolites), 14 plasma hormones, and the nine components of Levine's PhenoAge. Given the relatively the low sample size for multi‐omic experiments, we identified CR and EX multi‐omic signatures through a classification performance‐based approach rather than relying solely on multiple independent association tests, which could yield null results due to stringent corrections for multiple comparisons. Multi‐omic signatures for CR and EX were identified using Generalized Canonical Correlation Analysis (GCCA), which generalizes sparse Partial Least Squares (sPLS) regression to multiple omics datasets (Tenenhaus & Tenenhaus, 2011; Tenenhaus & Tenenhaus, 2014). This analysis was implemented in the R package *mixOmics*, following the “DIABLO” (Data Integration Analysis for Biomarker discovery using Latent variable for Omics studies) approach (Rohart, Gautier, et al., 2017).

We conducted two independent DIABLO analyses: one comparing CR to WD, and another comparing EX to WD, respectively. To identify the optimal set of multi‐omic biomarkers for the best classification performance in discriminating CR versus WD and EX versus WD, we performed five‐fold cross‐validation, repeated 100 times, using the *perf* function from the *mixOmics* R package (Rohart, Gautier, et al., 2017). This process identifies the best set of multi‐omic parameters by minimizing the balanced error rate (Rohart, Eslami, et al., 2017). The permutation procedure minimizes the type I error rate, enhances the robustness of the selected features, and avoids overfitting.

### 2.4 Characterization of the multi‐omic signatures: Enrichment analyses

We performed functional characterization of the multi‐omic features selected through the DIABLO method using enrichment analysis. Significantly enriched KEGG pathways were identified with the *multiGSEA* R package (Canzler & Hackermüller, 2020). The algorithm conducts separate overrepresentation tests for each omic layer and subsequently computes a combined p‐value using the Edgington method (Edgington, 1972). We chose the Edgington method over the more commonly used Fisher method for its more conservative nature, which helps to minimize type I error (Edgington, 1972), a critical consideration given the study's limited sample size. False discovery rate (FDR) correction for multiple testing was applied to further reduce the type I error rate. Four layers were included in this analysis: the gut microbiome metagenomics, the metabolome, the epigenome (DNAm) and the transcriptome (mRNA). For the gut microbiome, the selected OTUs were mapped to their corresponding KEGG orthologies and subsequently KEGG pathways using the PICRUSt2 software (Douglas et al., 2020), which predicts the functional potential of microbial communities by mapping OTUs to KEGG orthologs (KOs) and pathways through a multi‐step process: (1) OTU sequences are placed into a reference phylogeny; (2) these sequences are matched against a curated reference genome database; and (3) OTUs are mapped to KOs, translating microbial community composition into functional gene families linked to specific metabolic functions. KEGG pathways enriched in the DNAm signatures were identified using the *gometh* function from the *MissMethyl* R package (Phipson et al., 2016). This method accounts for two potential sources of bias in DNAm enrichment analysis: (1) the varying number of CpGs mapping within each gene and (2) the possibility of multiple annotations for a single CpG to more than one gene. Enriched KEGG pathways in the metabolome layer were identified using the *MetaboAnalystR* R package (Pang et al., 2021), where all compounds from the metabolomic analysis were cross‐referenced against the *MetaboAnalyst* libraries and annotated to the corresponding KEGG pathway. Overrepresentation analysis was performed using the hypergeometric test implemented in the R function *CalculateHyperScore*. Similarly, the list of genes in the RNAseq experiment were annotated to the corresponding KEGG pathways, with enrichment analysis conducted using the hypergeometric test from the *KEGGREST* R package and the *enrichKEGG* function (Nursalam, 2016 & Fallis 2013).

### 2.5 Enrichment of features in inflammatory pathways

In addition to the agnostic KEGG pathway enrichment analysis, we performed a targeted enrichment analysis focused specifically on inflammatory pathways, guided by literature suggesting that the potential anti‐aging effects of CR and EX may primarily be mediated through inflammation‐related pathways (Saavedra et al., 2023). This analysis utilized data from both the DNAm and mRNA layers. We clustered inflammation‐related genes according to the 17 inflammatory pathways described by the review by Loza et al. (Loza et al., 2007). Over‐representation tests for features pertaining to each pathway were conducted separately for DNAm and mRNA. For each pathway, we derived the empirical distribution of inflammatory features expected under the null hypothesis of no association via 10,000 permutations. The empirical p‐value was obtained by comparing observed versus expected distribution using multivariate hypergeometric test (Childs & Balakrishnan, 2000). As previously described, results from different omic layers were combined using the Edgington method (Edgington, 1972). This procedure includes a permutation‐based correction for multiple testing.

## 3 RESULTS

### 3.1 Lower values of multi‐omic BioAge biomarkers in CR and EX compared to WD

**FIGURE 1:**
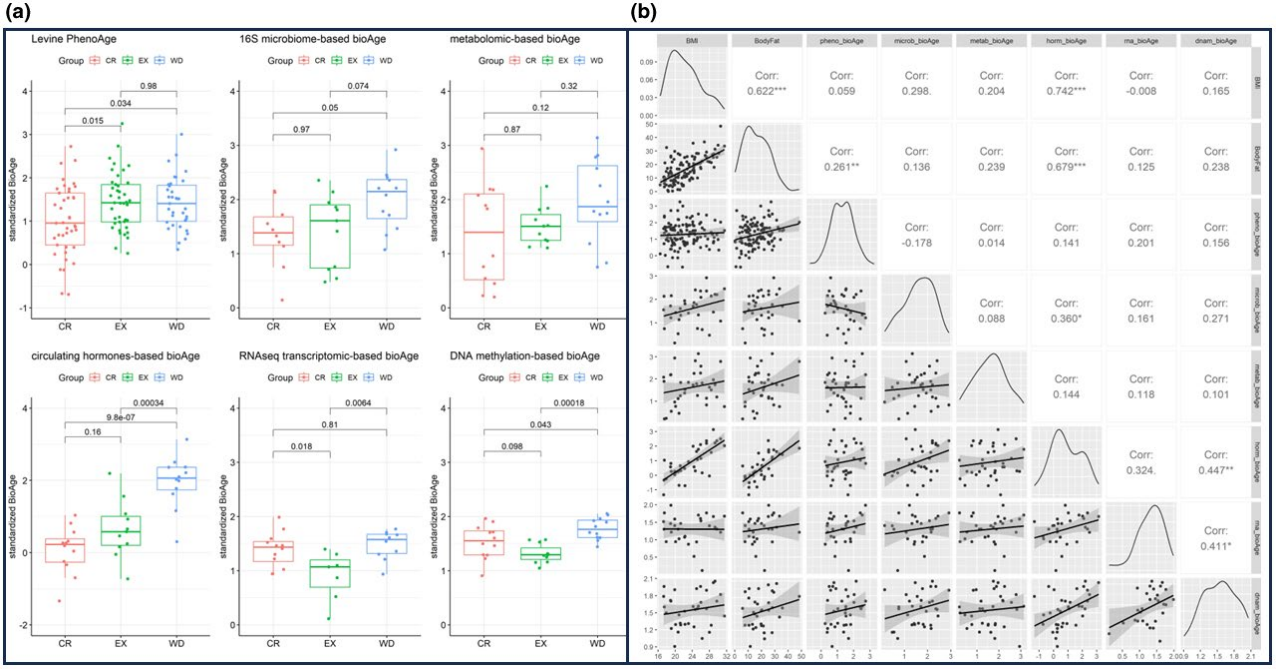
Comparative analysis of bioage measures and correlations with body composition metrics in calorie restriction, endurance exercise, and western diet groups. (a) Boxplots illustrating the six BioAge measures across the CR, EX, and WD groups. All *p*‐values have been adjusted for multiple comparisons using the Tukey HSD method. (b) Correlation plot displaying the relationships between BMI, body fat, and the six BioAge measures. The upper triangle shows pairwise Pearson correlation coefficients, the diagonal presents standardized distributions of each biomarker, and the lower triangle features pairwise scatterplots for each combination of measures.

A comparative analysis of the BioAge measures across the CR, EX, and WD groups revealed significant differences (Figure 1a). Notably, individuals in the CR group exhibited significantly lower values than those in the WD group (adjusted *p* < 0.05) for phenoBioAge, microbiomeBioAge, DNAmBioAge, and hormoneBioAge. Similarly, the EX group showed significantly lower values than the WD group for DNAmBioAge, transcriptomeBioAge, and hormoneBioAge. Additionally, CR participants displayed lower phenoBioAge values in comparison to EX participants, while endurance EX participants exhibited lower DNAmBioAge values relative to CR practitioners.

### 3.2 Comparison of BioAge biomarkers with BMI and body fat

To explore the relationships between BMI and body fat (assessed via Dual X‐ray Absorptiometry), and the six BioAge measures, pairwise Pearson correlation coefficients were calculated (Figure 1b). Significant correlations were observed between BMI and hormoneBioAge, as well as between body fat and both phenoBioAge and hormonesBioAge. Additionally, hormonesBioAge, DNAmBioAge, and transcriptomeBioAge demonstrated mutual correlation. However, the other BioAge measures did not reveal any significant correlation patterns.

### 3.3 Sensitivity analyses

For sensitivity analyses, we investigated three additional biomarkers: the “original” Levine's phenoAge, the Horvath epigenetic clock, and the epigenetic mutation load (EML), using DNAm data from colon mucosa (Gentilini et al., 2015; Horvath, 2013; Levine et al., 2018). These widely used biomarkers in aging research provide age estimates in years, thereby enhancing interpretability. However, they do have limitations, including susceptibility to batch effects and an inability to fully capture nonlinear relationships between aging and biomarker.

Our analyses revealed significant differences in PhenoAA—defined as the residuals of the linear regression of PhenoAge on chronological age—between the CR and WD groups (−2.96 years, *p* = 0.003) with no significant differences comparing EX with WD. Similarly, the Horvath DNAmAA was significantly lower in the EX group compared to the WD group (−2.25 years, *p* = 0.04) but not significantly different in the CR versus WD comparison. Furthermore, the EML age acceleration measure (EML_AA) showed significant lower values in the EX group relative to both the WD (−4.91 years, *p* = 0.003) and CR groups (−4.04 years, *p* = 0.02).

### 3.4 Multi‐omic signature of CR and EX

**FIGURE 2:**
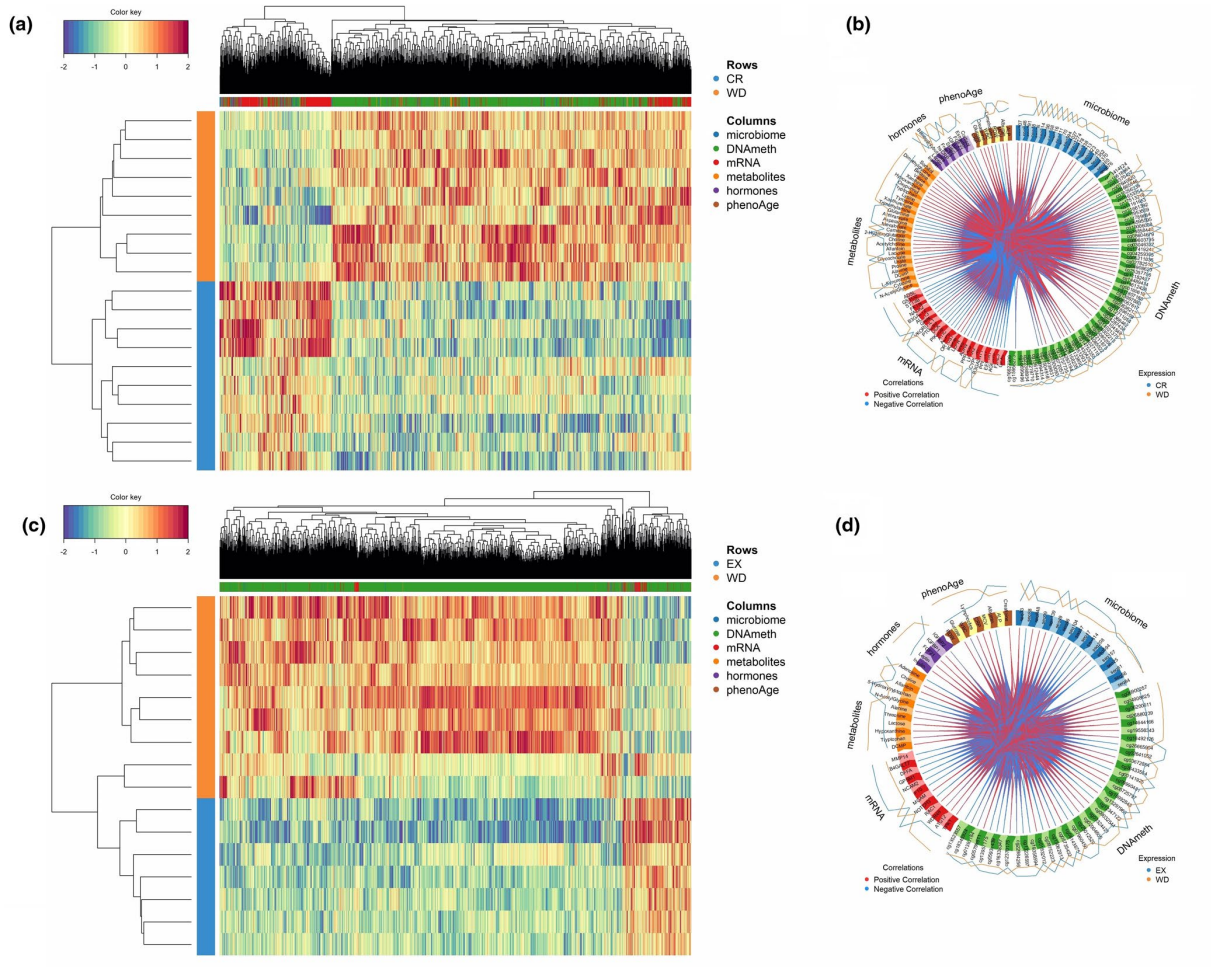
Multi‐omic signatures of calorie restriction and endurance exercise. (a) Heatmap illustrating the multi‐omic signature identified in the comparison of CR versus WD across various omic layers. (b) Circos plot depicting the correlation structure among the biomarkers constituting the multi‐omic CR signature. (c) Heatmap illustrating the multi‐omic signature identified in the comparison of EX versus WD across various omic layers. (d) Circos plot highlighting the correlation structure among the biomarkers in the multi‐omic EX signature.

Our multi‐omic discrimination analyses identified distinct multi‐omic signatures for both CR and endurance EX, as determined by comparing CR versus WD and EX versus WD, respectively. The multi‐omic signature associated with CR comprised 91 gut microbiome features, DNAm data from 1363 CpG sites, mRNA expression of 563 transcripts, 40 blood metabolites, and nine blood hormones (Figure 2a,b). In parallel, the multi‐omic signature for EX included 36 gut microbiome features, DNAm data from 2883 CpG sites, mRNA expression of 146 genes, 16 blood metabolites, and five blood hormones (Figure 2c,d). Detailed annotation of the selected features is available in the tables (S1–S10) where features exhibiting lower values in CR or EX compared to the WD are highlighted in blue, while those with higher values in CR or EX than WD are shown in red. A comparative analysis of the two signatures identified shared features: 16 microbial features, 71 CpG sites, four genes, 16 metabolites, and four hormones. Notably, the hypergeometric test confirmed that the observed overlap between the two signatures was significantly greater than what would be expected by chance (*p* < 0.0001).

Additionally, we conducted discrimination analyses between the CR and EX groups. However, this analysis failed to accurately classify CR and EX subjects into their respective groups based on cross‐validation. This indicates that the selected features cannot be considered robust biomarkers for distinguishing between these two classes. Consequently, we did not proceed with further characterization of this signature through pathway enrichment analysis.

### 3.5 KEGG pathways enrichment analysis

**FIGURE 3:**
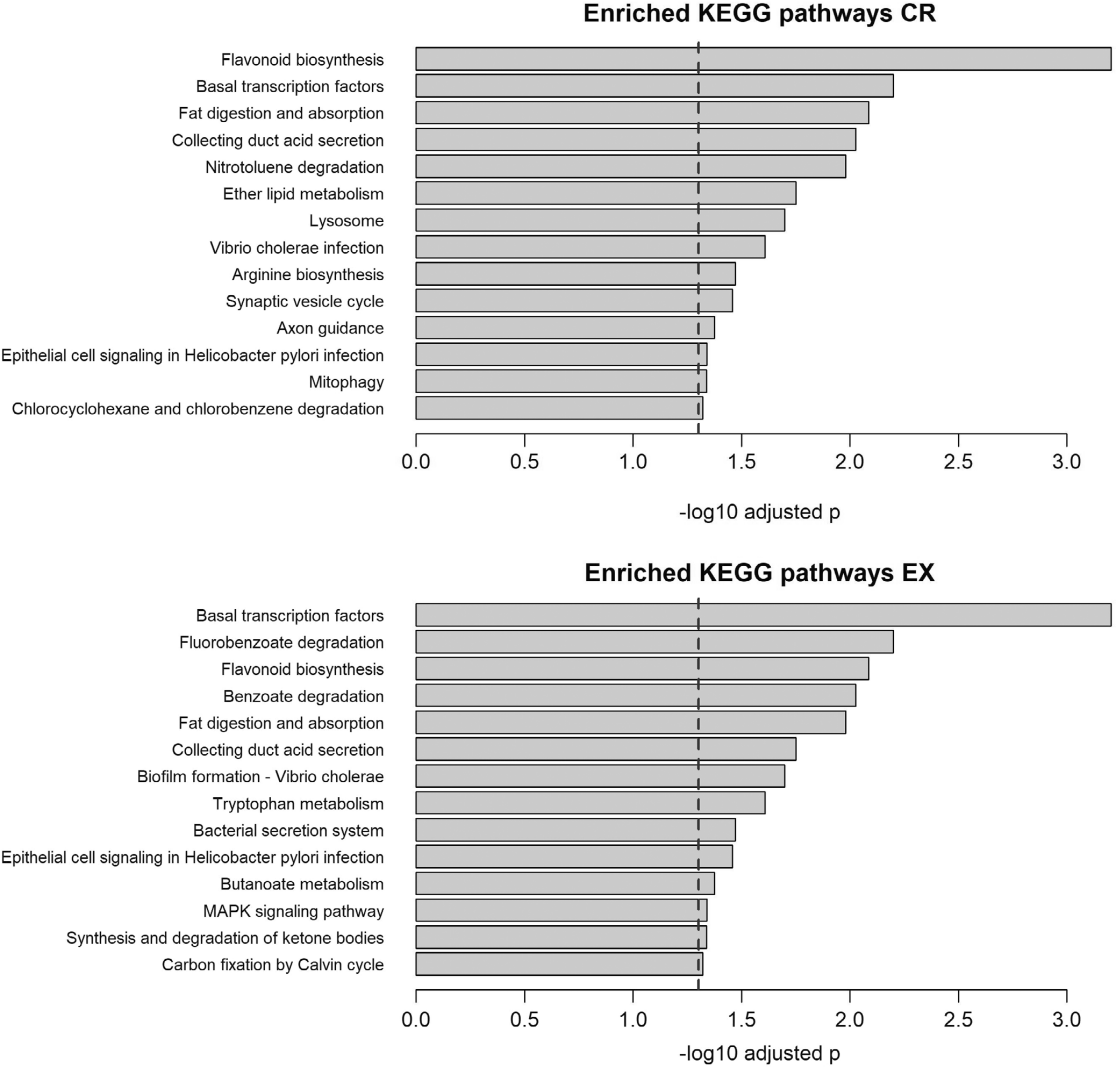
Enrichment of KEGG Pathways in CR and EX Signatures. Bar plots displaying the significantly enriched KEGG pathways in the comparison of CR versus WD (top) and in the comparison of EX versus WD (bottom). Enrichment analyses are based on four omic layers: Gut microbiome metagenomics, metabolomics, epigenomics (DNAm), and transcriptomics (mRNA). Pathway names are presented on the y‐axis, while FDR‐adjusted p‐values (−log10 transformed) are shown on the x‐axis.

The KEGG pathway enrichment analysis based on four layers (gut microbiome metagenomics, metabolomics, epigenomics, and the transcriptomics) identified 14 significantly enriched pathways, in the CR signature, as well as 14 pathways significantly enriched in the EX signature (adjusted *p* < 0.05). Notably, five pathways were common to both analyses: “*Flavonoid* biosynthesis,” “Basal transcription factors,” “Fat digestion and absorption,” “Collecting duct acid secretion,” and “Epithelial cell signalling in Helicobacter pylori infection” Furthermore, the analysis revealed nine pathways uniquely enriched in the CR signature and another nine pathways exclusively enriched in the EX signature. These findings are summarized in Figure 3, highlighting the distinctive and shared biological mechanisms associated with CR and EX.

### 3.6 Candidate inflammatory pathways enrichment analysis

**FIGURE 4:**
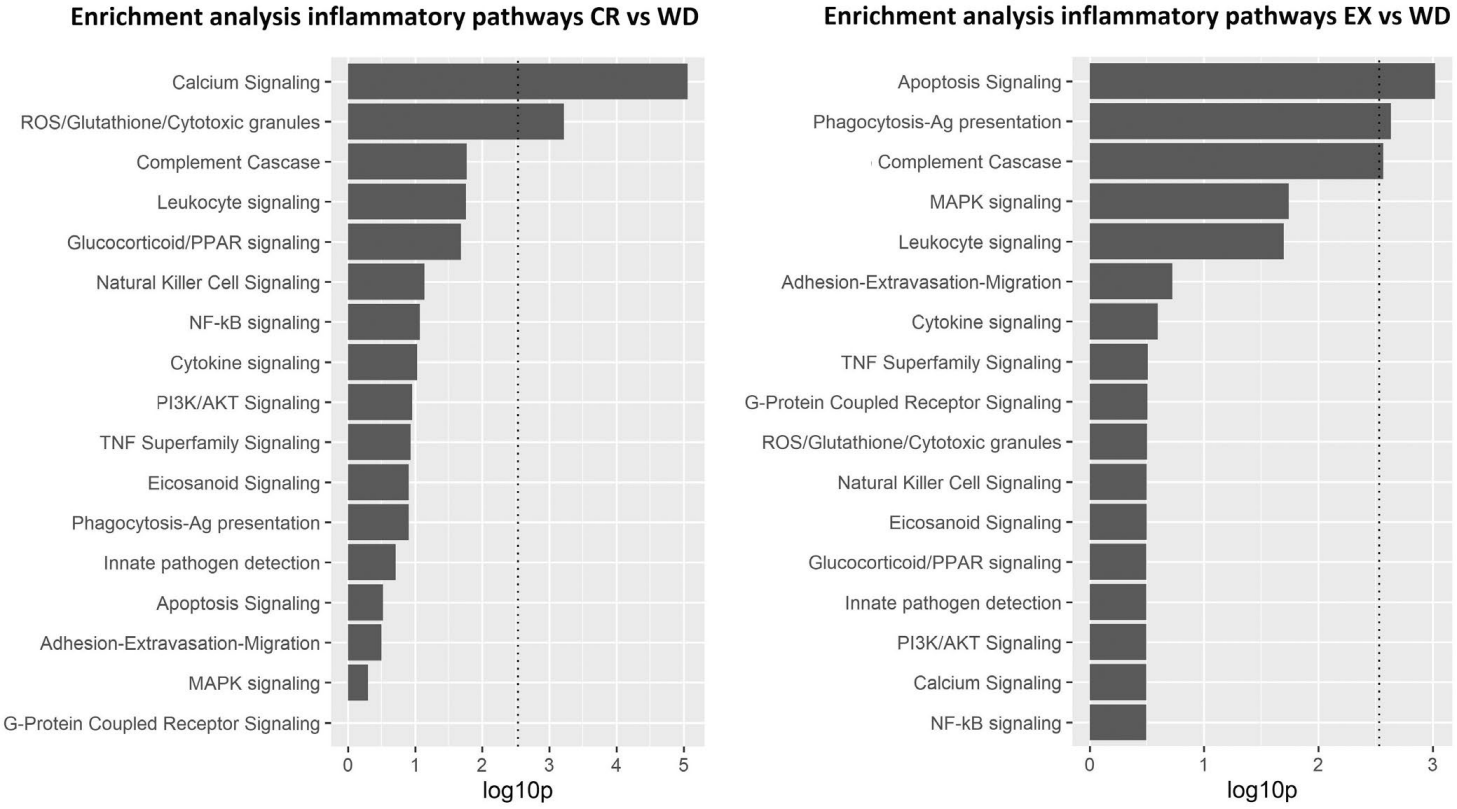
Enrichment of Inflammatory in CR and EX Signatures. Bar plots displaying the significantly enriched inflammatory pathways in the comparison of CR versus WD (left) and in the comparison of EX versus WD (right). Enrichment analyses are based on epigenomics (DNAm), and transcriptomics (mRNA) layers. Pathway names are presented on the y‐axis, while FDR‐adjusted *p*‐values (−log10 transformed) are shown on the x‐axis.

The results of the enrichment analyses of candidate inflammatory pathways based on DNAm and mRNA omic layers are summarized in Figure 4. Our findings revealed that the “Calcium signalling” and “Reactive oxygen species (ROS)/Glutathione/Cytotoxic granules” pathways were significantly enriched in the CR signature (empirical *p* < 0.0001 and *p* = 0.001, respectively). In contrast, the “*Apoptosis signalling, Phagocytosis‐Antigen presentation*,” and ‘*Complement cascade’* pathways were significantly enriched in the EX signature (*p* < 0.05). These results indicate a potential differential inflammatory response associated with CR and EX, suggesting a potential synergistic mechanism in modulating inflammation.

## 4 DISCUSSION

This study examined the effects of long‐term caloric restriction (CR) with adequate nutrition and EX training on biological age biomarkers, unveiling distinct multi‐omic signatures associated with these lifestyle interventions. Our findings revealed that both CR and EX groups exhibited significantly lower levels of multiple omic biomarkers of biological aging compared to sedentary controls consuming a Western diet. Specifically, CR participants showed lower phenoBioAge, microbiomeBioAge, and hormoneBioAge compared to their WD counterparts, which aligns with existing literature linking metabolic health and biological aging to dietary patterns (Green et al., 2022; Longo & Anderson, 2022). While our results do not definitively prove that CR and EX slow biological aging, they suggest potential aging mitigating effects, given the established correlation between these BioAge biomarkers and reduced mortality, as well as a lower risk of age‐related diseases (Moqri et al., 2024).

The significant lower levels of several biological age markers among CR participants compared to those on a Western diet align with findings from existing literature (Green et al., 2022; Levine et al., 2020; Maegawa et al., 2017). Preclinical data indicate that CR not only increases both average and maximal lifespan (Fontana et al., 2010; Ingram & de Cabo, 2017), but also mitigates age‐related diseases in both rodents and humans through mechanisms such as improved insulin sensitivity (Kraus et al., 2019; Weiss et al., 2007), reduced inflammation and oxidative stress (Il'yasova et al., 2018; Meydani et al., 2016), and the modulation of key nutrient sensing pathways (Fontana et al., 2018; Mercken et al., 2013; Yang et al., 2016). Furthermore, the finding that CR participants exhibited lower phenoBioAge compared to EX participants aligns with previous studies on life‐long rodents and humans showing that while EX improves health span and protects against fat accumulation and insulin resistance more effectively than CR, its impact on maximizing lifespan is less pronounced compared to that of CR. (Holloszy, 1997; Lee et al., 2022; Reimers et al., 2012). Specifically, the lower values in phenoBioAge among CR individuals points to the intervention's impact on metabolic pathways that are associated with biological aging processes (Levine et al., 2018).

Interestingly, in our study, master athletes exhibited a younger phenotype in DNAmBioAge and transcriptomeBioAge, both derived from colon mucosa biopsies, closely resembling that of younger WD individuals, potentially mediated by exercise‐induced anti‐inflammatory and senolytic response (Demaria et al., 2023; Makarewicz et al., 2022; Valenzuela et al., 2023). In contrast, CR practitioners exhibited lower BioAge values in gut microbiome‐ and blood‐derived measures, highlighting potential tissue‐specific differences in biological aging across interventions. Previous research has demonstrated that CR can lead to significant alterations in the gut microbiome and systemic metabolism (Griffin et al., 2017; Muegge et al., 2011), which may account for the observed lower values of CR than EX and WD in microbiomeBioAge.

Sensitivity analyses using additional—reference free—biological aging biomarkers, such as Levine's phenoAge, Horvath's epigenetic clock, and epigenetic mutation load (EML), reinforced the robustness of our findings and addressing potential bias due to the low sample size in the reference population (yWD) used for calculating the Mahalanobis distances.

Despite some limitations due to the small sample size of this study, we performed an exploratory analysis to identify multi‐omic signatures in CR and EX. This revealed two distinct signatures for each intervention, with more shared features than those expected by chance, suggesting both overlap and distinct biological pathways, consistent with prior studies (Broskey et al., 2019; Fiorito et al., 2021). Notably, pathways related to ‘*flavonoid biosynthesis*’ and ‘*basal transcription factors*’ emerged as key biological mechanisms shared between CR and EX signatures. Moreover, our findings regarding candidate inflammatory pathways revealed significant enrichment of genes related to the ‘*reactive oxygen species (ROS)/glutathione/cytotoxic granules*’ pathway in the CR signature, underscoring the importance of oxidative stress‐related mechanisms in CR. This aligns with findings from other studies that demonstrate the potent antioxidant and anti‐inflammatory effects of CR (Davì et al., 2002; Il'yasova et al., 2018), both in primary and secondary prevention contexts (Larson‐Meyer et al., 2006; Meydani et al., 2016; Ruggenenti et al., 2017).

The shared ‘*basal transcription factor*’ KEGG pathway in CR and EX may indicate that both interventions counteract aging by reducing stochastic (possibly epigenetic) DNA damage, particularly affecting transcription factors like the TFII complex, which are critical to age‐related gene expression changes (Frenk & Houseley, 2018).

Among the KEGG pathways uniquely enriched in the CR signature, ‘*ether lipid metabolism*’ is particularly notable. Studies in *C. elegans* link genes in this pathway to longevity, suggesting ether lipids play a key role in aging (Cedillo et al., 2023). In humans, centenarians often exhibit elevated levels of alkyl ether lipids, which are more resistant to lipid peroxidation, offering protection against oxidative damage (Pradas et al., 2019). These findings highlight reducing damage due to oxidative stress as a critical factor in healthy aging and suggest targeting ether lipid metabolism could be a promising therapeutic approach for anti‐aging interventions.

This study's limitations primarily stem from its cross‐sectional design, which restricts the ability to establish causal relationships. While randomized clinical trials would provide ideal confirmation, conducting long‐term (>3 years) CR and exercise training trials presents challenges related to feasibility, adherence and cost. Additionally, the small reference population of young individuals (yWD) may affect the accuracy of the Mahalanobis distance used to determine biological age (BioAge), as obtaining colon biopsies from this demographic is inherently difficult. Despite these challenges, sensitivity analyses employing alternative biomarkers—Levine's PhenoAge and Horvath's epigenetic clock, which do not rely on a reference population—produced consistent results, thereby enhancing our confidence in the findings. Another limitation of the study is the small percentage of women in the sample (less than 20%), which may impact the generalizability of our results. Unique aspects of this research include the focus on participants engaged in chronic CR with optimal nutrition and high‐volume EX over extended periods—conditions difficult to replicate in typical randomized intervention trials. Moreover, the sedentary control group displayed no evidence of chronic diseases or metabolic disorders. A key strength of this study is its use of multiple omic measurements—including colon mucosa tissue, DNAm, gene expression, gut microbiome, and blood metabolomics and hormones—yielding consistent results across different omic domains, despite weak correlations among the six BioAge measures. Although the study has low statistical power, significant differences were observed across various tissues and omic types, suggesting that these findings are meaningful and substantial despite the study's limitations. However, the study's small sample size limits the generalizability and robustness of the findings, hindering definitive conclusions about the effects of CR and EX while also restricting control over confounding factors like dietary quality, education, and lifestyle. This limitation also precludes investigation of a dose–response relationship, as the duration of CR correlates strongly with chronological age, complicating the differentiation between dose‐dependent and age‐related effects. Additionally, we could not assess the potential mediating effects of BioAge biomarkers on clinically relevant health outcomes such as cognitive function or disease risk profiles. Future research with larger cohorts and tailored designs is essential to accurately evaluate these relationships. Furthermore, the inability to split the dataset into training and test sets for multi‐omic signature identification necessitates cautious interpretation of these findings, which, while supported by existing literature, require further validation.

In conclusion, our pilot study offers compelling evidence that humans adhering to CR with adequate nutrition or engaged in EX have significant lower levels of multi‐omic BioAge biomarkers than sedentary controls consuming on a Western diet. Notably, the reduction of oxidative stress related damage emerges as a possible anti‐aging mechanism shared by both interventions. These results support existing evidence about the critical role of lifestyle modifications in promoting longevity and reducing the risk of age‐related diseases. Further research is essential to elucidate the molecular mechanisms underlying the anti‐aging effects of CR and EX, and to validate these findings through randomized clinical trials.

## AUTHOR CONTRIBUTIONS

LF designed and conceived the study. GF and SP conducted the bioinformatics and biostatistical analyses. VT, BB, NV, EC, and FS collected biological samples, extracted serum, DNA, and RNA, and performed the multiomic wet lab measurements. DSE, DR and LF supervised the wet lab experiments. PV and LF supervised the statistical analyses and interpretation of the results. All authors contributed to the drafting and editing of the manuscript.

## FUNDING INFORMATION

L.F. is supported by grants from the Australian NHMRC Investigator Grant (APP1177797), Australian Youth and Health Foundation, and Bakewell Foundation. G.F. is funded by the Italian Ministry of Health through 5×1000 and ‘Ricerca Corrente’ at IRCCS Istituto Giannina Gaslini. S.P. is supported by the project NODES, which has received funding from the Italian Ministry of University and Research– M4C2 1.5 of PNRR with the grant agreement no. ECS00000036.

## CONFLICT OF INTEREST STATEMENT

The authors have no financial conflicts of interest to disclose.

## Supporting information


Table S1.
**Table S2**.
**Table S3**.

Data S1.

## Data Availability

Data used in this study will be available upon request to the corresponding author.
