# Supplementary material for: Multi‐omic analysis of biological aging biomarkers in long‐term calorie restriction and endurance exercise practitioners: A cross‐sectional study
Source: Aging Cell. 2024 Dec 18;24(4):e14442. doi: 10.1111/acel.14442 (PMC11984672; doi:10.1111/acel.14442)
Supplement: Supplementary file 2 — Data S1. [file ACEL-24-e14442-s001.docx]

**Online methods**

Study population

This study recruited three groups of volunteers, referred to as calorie restriction (CR), endurance exercise (EX), and Western diet (WD). The CR group comprised 41 well-characterized, weight-stable, middle-aged and very lean men and women who had practiced CR for a maximum of 15 years while ensuring adequate nutrition (at least 100% of the Reference Daily Intake -RDI- for each nutrient). The second group consisted of 41 master athletes who engaged in running (at least 30 miles per week) or expended equivalent energy through cycling or swimming for at least the past three years. Participants in the EX group were matched with the CR group based on age and sex. The third group (WD) comprised of 35 sedentary controls (defined as engaging in regular exercise for less than 1 hour per week), also matched for age and sex, who consumed a typical Western diet and were recruited from the St. Louis area. All participants had no evidence of chronic diseases, were non-smokers, and did not take medications that could influence the outcome variables. Weight stability was defined as less than a 2-kg change in body weight over the previous six months. Each participant recorded their food and beverage intake for seven consecutive days, which was analyzed using the NDS-R software program (version 2012, v.4.03_31). Height and body weight measurements were taken in the morning after an overnight fast while participants wore only underwear and a hospital gown. Total body fat mass and fat-free mass were determined using dual-energy X-ray absorptiometry (DXA; QDR 1000/w; Hologic).

Multi-omic characterization

*Gut microbiome*

DNA was isolated from aliquots of pulverized frozen human feces as described in Griffin et al. (Griffin et al. 2017). The V4 region of bacterial 16S rRNA genes present in fecal samples was amplified by PCR and sequenced using an Illumina MiSeq instrument, according to protocols detailed in Bokulich et al. (Bokulich et al. 2013). Paired reads were trimmed to 200 bases and assembled them using FLASH (v1.2.11) with default parameters, plus a minimum overlap of 18 bases and a maximum overlap of 125 bases. Reads sharing at least 97% nucleotide sequence identity with a reference sequence in the GreenGenes 16S rRNA database (DeSantis et al. 2006) were assigned to that operational taxonomic unit (OTU). Unassigned sequences were subsequently grouped into de novo OTUs. OTUs were assigned taxonomy using the RDP classifier v2.4 (Wang et al. 2007). Before analysis, we removed OTUs that failed sequence alignment by PyNast (Caporaso et al. 2010). To be retained in the dataset, OTUs had to satisfy at least one of the following conditions: (i) made up at least 0.05% of the reads in at least one sample, (ii) was detected in at least 1% of human donor fecal samples, (iii) made up at least 0.1% of the reads from at least one sample.

*DNA methylation (colon mucosa)*

Biopsy specimens of normal-appearing sigmoidal colon mucosa were collected from all the volunteers in the morning after an overnight fast and a preparation with an enema containing water. Colonic mucosal specimens were immediately washed in PBS and then flash-frozen in liquid nitrogen and stored at -80°C until processed. Genomic DNA was isolated as per the standard DNA extraction procedure using the DNeasy Tissue Kit (Qiagen, Hilden Germany). DNA methylome profiling was carried out using Illumina HumanMethylation450 BeadChip. Data pre-processing was carried out using in-house software written for the R statistical computing environment described previously (Gagliardi et al. 2020). For each sample and probe, measurements were set to missing if obtained by averaging intensities over less than three beads or if averaged intensities were below detection thresholds estimated from negative control probes. Background subtraction and dye bias correction (for probes using the Infinium II design) were also performed. The probes close to SNPs with minor allele frequency greater than 0.05 in Europeans and potentially cross-reactive probes were removed (Pidsley et al. 2016). Additionally, probes detected in < 20% of the samples were excluded from the analyses leaving us with N = 346,699 for the analyses. Methylation levels at each CpG locus were expressed as the ratios of intensities arising from methylated CpGs over those arising from the sum of methylated and unmethylated CpGs (beta values).

*RNA sequencing (colon mucosa)*

Total RNA integrity was determined using Agilent Bioanalyzer or 4200 Tapestation. Library preparation was performed from a starting input of 10ug of total RNA. Ribosomal RNA was removed by poly-A selection using Oligo-dT beads (mRNA Direct kit, Life Technologies). mRNA was then fragmented in reverse transcriptase buffer and heated to 94 degrees for 8 minutes. mRNA was reverse transcribed to yield cDNA using SuperScript III RT enzyme (Life Technologies, per manufacturer’s instructions) and random hexamers. A second strand reaction was performed to yield ds-cDNA. cDNA was blunt ended, had an A base added to the 3’ ends, and then had Illumina sequencing adapters ligated to the ends. Ligated fragments were then amplified for 12-15 cycles using primers incorporating unique dual index tags. Finally, fragments were sequenced on an Illumina HiSeq3000 using single end reads extending 50 bases.

*Plasma Metabolomics*

Metabolomics analyses were performed at the University of Washington Medicine by Dr Raftery. A commercially available pooled serum sample (Innovative Research, Novi, MI, USA) was used as quality control (QC). As previously described, both QC and study samples were prepared at The Northwest Metabolomics Research Center following a standard operating protocol (Parent et al. 2016; Buas et al. 2016). Targeted LC-MS/MS experiments were performed on an Agilent 1260 LC (Agilent Technologies, Santa Clara, CA) AB Sciex QTrap 5500 MS (ABsciex, Toronto, Canada) system, controlled by Analyst 1.5 software. Two samples injection (5 uL and 10 uL) were used for LC-MS/MS analysis in positive and negative ion models, respectively. Replicates of two independent QC serum samples were injected after every 10 study samples. Chromatographic separations were performed in hydrophilic interaction chromatography (HILIC) mode. The flow rate and column temperature were set to 300 uL/min and 40º C, respectively. Targeted data acquisition was performed in multiple-reaction-monitoring (MRM) mode. The extracted MRM peaks were integrated using MultiQuant 2.1 software (AB Sciex, Toronto, ON, Canada).

*Blood Hormones*

Venous blood samples were obtained after subjects had fasted for at least 12 hours overnight. Serum and plasma samples were aliquoted and frozen at -80°C and tested at the end of the Study, batched so that all samples were included in the same assay.

Plasma Insulin, Cortisol, Free Triiodothyronine (FT3), Sex hormone-binding globulin (SHBG), Estradiol and Testosterone were measured by ECLIA electrochemiluminescence (Elecsys Roche Diagnostic, Lewes England, on the Roche cobas e601). High-sensitivity C-reactive protein (hsCRP) was run by a particle enhanced immunoturbidimetric essay using Roche reagents on the Roche cobas c501. Insulin-like Growth Factor 1 (IGF-1) and Insulin-like Growth Factor Binding Protein 3 (IGFBP-3) were run using chemiluminescence with Siemens kits on Siemens Immulite 1000. Insulin-like Growth Factor Binding Protein 1 (IGFBP-1) was run by ELISA using Alpco kits. Commercial ELISA Quantikine kits from R&D System Inc, Minneapolis, MN were used to measure Fibroblast Growth Factor 21 (FGF21) and High Molecular Weight (HMW) Adiponectin. Leptin was run by RIA using Millipore kits. All the measurements were performed at the Core Laboratory for Clinical Studies at the Washington University in St.Louis.

Statistical analyses

*Computation of the omicBioAge measures*

After preprocessing and quality controls we analyzed:

- nine blood and immune system biomarkers (Albumin, Creatinine, Glucose, CRP, Lymphocyte percentage, mean cell volume -MCV-, red cell distribution width -RDW-, alkaline phosphatase -ALP-, white blood cell count -WBC-) composing the phenoBioAge,
- 1,321 OTUs (microbiomeBioAge),
- 346,699 CpG sites (DNAmBioAge),
- 14 hormones (Adiponectin, BHB, Cortisol, Estradiol, Testosterone, SHBG, FGF21, FreeT3, hsCRP, IGF1, IGFBP1, IGFBP3, Leptin, Insulin) composing the hormoneBioAge,
- 23,562 transcripts (transcriptomeBioAge),
- 108 blood metabolites (metabBioAge).

For each set of omic biomarkers, we calculated the individual's BioAge based on the definition of Homeostatic Dysregulation (HD) as described by Cohen (Cohen 2016). The HD value reflects individuals’ degree of deviation of their global omic profile compared to a reference (healthy) population. Higher HD values indicate a higher distance from the reference population, that is interpretable as a more advanced state of biological aging correlated with a higher risk of ageing related diseases and mortality. Contrarily, lower values of HD suggest a slower pace of biological aging, potentially reducing the risk of adverse health outcomes. As a reference population, we utilized six young individuals following a Western Diet, with ages ranging from 21 to 27 and an average age of 24 (yWD).

HD is determined using the Mahalanobis distance (De Maesschalck et al. 2000) applied to each set of biomarkers separately, to derive the six omic BioAge measures. The Mahalanobis distance equation is formulated as follows:

$$\mathrm{HD}\left( x \right)= \sqrt{\sum_{i=1}^{n} \frac{{{(x}_{i}-\mu_{i})}^{2}}{\sigma^{2}(x_{i})}}$$

where **x** is a multivariate observation (all the feature values for an individual), **μ_i_** is the average value for the i-th feature in the reference population (yWD), **n** is the number of biomarkers and **σ^2^(x_i_)** is the variance in the i-th feature. For the analysis, all the biomarkers were standardized to unit standard deviation keeping the average value for the reference group (yWD) to zero. The six BioAge measures were further standardized accommodating variations in the number of features per omic, allowing for meaningful comparisons.

Sensitivity analyses: PhenoAgeAA, epigenetic clocks, and epigenetic mutation load

For sensitivity analyses, we investigate three additional biomarkers: the 'original' Levine’s phenoAge measure, Horvath epigenetic clock, and the epigenetic mutation load. Below we provide a description of methods for computing the three additional BioAge markers and the results for the comparison across the three groups.

*Phenotypic age (blood PhenoAge)*

Blood phenotypic age (PhenoAge) was calculated as previously described by Levine et al. (Levine et al. 2018). Briefly, PhenoAge is a composite score including chronological age and the nine blood measured biomarkers listed in previous section. These nine biomarkers together with chronological age were included in a parametric proportional hazards model based on the Gompertz distribution. Based on this, authors estimated the 10-year mortality risk for each individual in the training test. Next, the mortality score was converted into units of years via inverse formula (PhenoAge). Phenotypic age acceleration (PhenoAA) was defined as the residuals of PhenoAge on chronological age. Positive values of PhenoAA (i.e., phenotypic age higher than the chronological age) indicate accelerated aging and vice versa.

*Horvath epigenetic clock (colon mucosa DNAmAge)*

DNA methylation age (DNAmAge) was computed according to the algorithm described by Horvath (Horvath 2013), as a linear combination of 353 age-associated CpG sites, with weights described in the original publication. Epigenetic age acceleration (DNAmAA) was defined as the residuals of DNAmAge on chronological age. Similarly to PhenoAgeAA, positive values of DNAmAA indicate accelerated aging and vice versa.

*Epigenetic mutation load (DNAm colon mucosa)*

Epigenetic mutation load (EML) is defined as the sum of stochastic epigenetic mutations (SEM), according to Gentilini et al. (Gentilini et al. 2015). In short, for each CpG site, extreme values were determined as those whose DNAm levels fell outside the range constituted by the first quartile (Q1) minus three times the interquartile range -IQR- (lower boundary), and the third quartile (Q3) plus three times the IQR (upper boundary). These extreme values are defined as SEMs, and the sum of SEMs for each individual is defined as the EML. For the analyses we compute the natural logarithm of (EML). For comparison with PhenoAge and DNAmAge, EML data were rescaled to be expressed in years as described in Fiorito et al. (Fiorito et al. 2019). Consistently, we computed EML acceleration (EML_AA) as the residual of EML regression on chronological age.

**Supplementary references**

Bokulich NA, Ohta M, Richardson PM & Mills DA (2013) Monitoring Seasonal Changes in Winery-Resident Microbiota. *PLoS One*.

Buas MF, Gu H, Djukovic D, Zhu J, Drescher CW, Urban N, Raftery D & Li CI (2016) Identification of novel candidate plasma metabolite biomarkers for distinguishing serous ovarian carcinoma and benign serous ovarian tumors. *Gynecol. Oncol.*

Caporaso JG, Bittinger K, Bushman FD, Desantis TZ, Andersen GL & Knight R (2010) PyNAST: A flexible tool for aligning sequences to a template alignment. *Bioinformatics*.

Cohen AA (2016) Complex systems dynamics in aging: new evidence, continuing questions. *Biogerontology* 17.

DeSantis TZ, Hugenholtz P, Larsen N, Rojas M, Brodie EL, Keller K, Huber T, Dalevi D, Hu P & Andersen GL (2006) Greengenes, a chimera-checked 16S rRNA gene database and workbench compatible with ARB. *Appl. Environ. Microbiol.*

Fiorito G, McCrory C, Robinson O, Carmeli C, Rosales CO, Zhang Y, Colicino E, Dugué P-A, Artaud F, McKay GJ, Jeong A, Mishra PP, Nøst TH, Krogh V, Panico S, Sacerdote C, Tumino R, Palli D, Matullo G, Guarrera S, Gandini M, Bochud M, Dermitzakis E, Muka T, Schwartz J, Vokonas PS, Just A, Hodge AM, Giles GG, Southey MC, Hurme MA, Young I, McKnight AJ, Kunze S, Waldenberger M, Peters A, Schwettmann L, Lund E, Baccarelli A, Milne RL, Kenny RA, Elbaz A, Brenner H, Kee F, Voortman T, Probst-Hensch N, Lehtimäki T, Elliot P, Stringhini S, Vineis P, Polidoro S, Alberts J, Alenius H, Avendano M, Baltar V, Bartley M, Barros H, Bellone M, Berger E, Blane D, Candiani G, Carra L, Castagné R, Chadeau-Hyam M, Cima S, Clavel-Chapelon F, Costa G, Courtin E, Delpierre C, D’Errico A, Dermitzakis M, Elovainio M, Elliott P, Fagherazzi G, Fraga S, Garès V, Gerbouin-Rerolle P, Giles GG, Goldberg M, Greco D, Guessous I, Haba-Rubio J, Heinzer R, Hodge AM, Joost S, Karimi M, Kelly-Irving M, Kähönen M, Karisola P, Khenissi L, Kivimaki M, Laine J, Lang T, Laurent A, Layte R, Lepage B, Lorsch D, MacGuire F, Machell G, Mackenbach J, Marmot M, de Mestral C, Miller C, Milne RL, Muennig P, Nusselder W, Petrovic D, Pilapil L, Preisig M, Pulkki-Råback L, Raitakari O, Ribeiro AI, Ricceri F, Recalcati P, Reinhard E, Valverde JR, Saba S, Santegoets F, Satolli R, Simmons T, Severi G, Shipley MJ, Tabak A, Terhi V, Tieulent J, Vaccarella S, Vigna-Taglianti F, Vollenweider P, Vuilleumier N & Zins M (2019) Socioeconomic position, lifestyle habits and biomarkers of epigenetic aging: A multi-cohort analysis. *Aging (Albany. NY).* 11, 2045–2070.

Gagliardi A, Dugué P-A, Nøst TH, Southey MC, Buchanan DD, Schmidt DF, Makalic E, Hodge AM, English DR, Doo NW, Hopper JL, Severi G, Baglietto L, Naccarati A, Tarallo S, Pace L, Krogh V, Palli D, Panico S, Sacerdote C, Tumino R, Lund E, Giles GG, Pardini B, Sandanger TM, Milne RL, Vineis P, Polidoro S & Fiorito G (2020) Stochastic Epigenetic Mutations Are Associated with Risk of Breast Cancer, Lung Cancer, and Mature B-cell Neoplasms. *Cancer Epidemiol. Biomarkers Prev.*

Gentilini D, Garagnani P, Pisoni S, Bacalini MG, Calzari L, Mari D, Vitale G, Franceschi C & Di Blasio AM (2015) Stochastic epigenetic mutations (DNA methylation) increase exponentially in human aging and correlate with X chromosome inactivation skewing in females. *Aging (Albany. NY).*

Griffin NW, Ahern PP, Cheng J, Heath AC, Ilkayeva O, Newgard CB, Fontana L & Gordon JI (2017) Prior Dietary Practices and Connections to a Human Gut Microbial Metacommunity Alter Responses to Diet Interventions. *Cell Host Microbe*.

Horvath S (2013) DNA methylation age of human tissues and cell types. *Genome Biol.*

Levine ME, Lu AT, Quach A, Chen BH, Assimes TL, Bandinelli S, Hou L, Baccarelli AA, Stewart JD, Li Y, Whitsel EA, Wilson JG, Reiner1 AP, Aviv1 A, Lohman K, Liu Y, Ferrucci L & Horvath S (2018) An epigenetic biomarker of aging for lifespan and healthspan. *Aging (Albany. NY).*

De Maesschalck R, Jouan-Rimbaud D & Massart DL (2000) The Mahalanobis distance. *Chemom. Intell. Lab. Syst.* 50.

Parent BA, Seaton M, Sood RF, Gu H, Djukovic D, Raftery D & O’Keefe GE (2016) Use of metabolomics to trend recovery and therapy after injury in critically ill trauma patients. *JAMA Surg.*

Pidsley R, Zotenko E, Peters TJ, Lawrence MG, Risbridger GP, Molloy P, Van Djik S, Muhlhausler B, Stirzaker C & Clark SJ (2016) Critical evaluation of the Illumina MethylationEPIC BeadChip microarray for whole-genome DNA methylation profiling. *Genome Biol.*

Wang Q, Garrity GM, Tiedje JM & Cole JR (2007) Naïve Bayesian classifier for rapid assignment of rRNA sequences into the new bacterial taxonomy. *Appl. Environ. Microbiol.*
